# Hypoglycemic activities of lyophilized powder of *Gynura divaricata* by improving antioxidant potential and insulin signaling in type 2 diabetic mice

**DOI:** 10.3402/fnr.v59.29652

**Published:** 2015-12-28

**Authors:** Bing-Qing Xu, Ping Yang, Yu-Qing Zhang

**Affiliations:** Silk Biotechnology Laboratory, School of Biology and Basic Medical Sciences, Soochow University, Suzhou, China

**Keywords:** diabetes, hypoglycemic, *Gynura divaricata*, insulin resistance, glucose metabolism, AKT/PI3K

## Abstract

**Background:**

Diabetes mellitus is a serious disease affecting about 5% of people worldwide. Although several studies have indicated hypoglycemic activities of *Gynura divaricata* (GD), the mechanisms by which GD improves the symptoms of diabetes remain unclear.

**Objective:**

The aim of this study was to investigate the potential hypoglycemic effects of GD.

**Design:**

The leaves and stems of GD were prepared and lyophilized into a powder, which was added to the diet of mice with type 2 diabetes induced by a high-fat diet in combination with streptozotocin for 4 weeks. During this period, fasting blood glucose (FBG) levels and body weight of mice were measured. In addition, at the end of the experiment, a series of assays was performed.

**Results:**

GD administration effectively alleviates insulin resistance and induces a decrease in FBG by 59.54% in 1.2% (L) GD-treated diabetic group and 56.13% in 4.8% (H) GD-treated diabetic group after 4 weeks, respectively, relative to diabetic model mice. The antioxidant capacity was improved by increasing the activities of glutathione peroxidase (GSH-Px) and total superoxide dismutase (T-SOD) by 64.87% and 53.42% in treatment group H, compared to diabetic model mice, while GD treatment induced a significant decrease in malondialdehyde (MDA) level by 50% in treatment group L, compared to the level in diabetic model mice. Furthermore, glucose metabolism was ameliorated by the increased glycogen synthesis in the livers of diabetic mice. In addition, we also demonstrated that the messenger RNA (mRNA) and protein expression levels of AKT, PI3K and PDK-1, which are involved in insulin signaling, were significantly increased.

**Conclusions:**

Oral administration of the GD-lyophilized powder has been effectively hypoglycemic, which is done by activating insulin signaling and improving antioxidant capacity in mice with type 2 diabetes.

Type 2 diabetes mellitus (T2DM), which is an epidemic globally, is also known as non-insulin-dependent diabetes mellitus, and it is characterized by insulin resistance and impaired insulin secretion due to beta-cell dysfunction (1). Insulin resistance is a state where insulin has less ability to mediate glucose homeostasis in its major target tissues such as liver, skeletal muscle and adipose tissue (2). For regulating blood glucose to a normal level, organisms secrete excessive insulin for compensation, which causes hyperinsulinemia. At present, synthetic drugs often cause some side effects. Therefore, some traditional Chinese medicinal herbs are emerging as good alternatives.

*Gynura divaricata* L. (GD) is a traditional Chinese medicinal herb, generally known as ‘Bai Bei San Qi’ in China (3), that belongs to the vegetable family. GD contains many natural components, including polysaccharides, flavonoids, organic acids, terpenoids, alkaloids, phenolic compounds, fatty acids, and cerebrosides (4–13). GD is widely used in the prescription of traditional Chinese medicine for the treatment of diabetes, hypertension and other diseases, including several tumors (14). It also enhances the function of gastrointestinal peristalsis (15). The hypoglycemic effect of GD was studied using insulin-resistant HepG2 cells that were treated with insulin for 36 h. The results showed that GD had no effect on the proliferation of HepG2 cells but significantly improved insulin resistance in these cells (16). In addition, GD extracts can inhibit α-amylase and α-glucosidase activity *in vitro* (17). The GD extract may have an antihypertensive effect in a spontaneous hypertensive rat model by decreasing the serum endothelin content and subsequently increasing nitric oxide (NO) content and superoxide dismutase (SOD) activity. It was obvious that the GD extract had a protective effect on the major organ damage caused by hypertension (18, 19). GD also demonstrated hepatoprotective activity by decreasing alanine glutamic oxaloacetic transaminase activity in the serum and reducing the degeneration and necrosis of liver cells (20).

This study aims to illustrate the underlying mechanism of the potential hypoglycemic effect of GD-lyophilized powder as a dietary additive in diabetic mice induced by a high-fat diet and streptozotocin (STZ).

## Materials and methods

### Materials and chemicals

The GD was obtained from Silk Biotechnology Laboratory, Soochow University (Suzhou, China). Fresh leaves and stems of GD were collected, washed and then lyophilized into a powder. The powder was stored in 4°C for further research. Reverse transcription (RT), real-time quantitative polymerase chain reaction (qPCR), and SYBR green I reagents were purchased from ShineGene Co. (Shanghai, China). Glucose standard, rutin, phenol, trichloroacetic acid and aluminum nitrate were provided by Chemical Reagent Co. Ltd., National Medicine Group (China).

### Determination of total polysaccharides

The total polysaccharides content of GD was determined as previously reported by Wang et al. (21), with slight modifications. The absorbance was measured at 490 nm. The glucose standard curve was drawn with the absorbance as the vertical axis (*y*) and the concentrations of the glucose (mg/mL) as the abscissa (*x*). We weighed 3 g of GD in 300 mL of water, which was at 121°C for high-pressure sterilization for 2 h. The sample was extracted three times. The combined extract was centrifuged at 7,000 r/min to obtain the supernatant. The supernatant was then concentrated and added into three volumes of 95% ethanol solution. The mixture was allowed to stand overnight at 4°C and then centrifuged at 11,000 r/min for 20 min. The precipitate was dissolved in 23 mL of distilled water and added to 10 mL of a 10% trichloroacetic acid solution to remove the protein. After being centrifuged at 7,000 r/min for 20 min, 1 mL of supernatant was diluted to 100 mL as a diluted sample solution and 1 mL of that solution was precisely added into a centrifuge tube. The remaining steps were repeated with the standard assay, and the absorbance was measured at a wavelength of 490 nm to calculate the polysaccharide content of GD sample. The thrice extracted solutions for each sample were then measured, and the standard deviation was calculated, represented by±SD.

### Determination of total flavonoids

The total flavonoids content of GD was determined as previously reported by Wang et al. (22). The absorbance was measured at 510 nm. The rutin standard curve was drawn with the absorbance as the vertical axis (*y*) and the concentrations of the rutin (mg/mL) as the abscissa (*x*). The linear regression equation was obtained using the least squared method according to the absorbance value and the amount of concentrations of the rutin established from the rutin standard curve. The GD sample (0.5 g) was dissolved in 50 mL of 80% ethanol and extracted for 3 h at 60°C. After the extracted solution was centrifuged by 4,000×g for 10 min at 4°C, 1 mL of the supernatant was precisely added into a centrifuge tube. The remaining steps were repeated with the standard assay, and the absorbance was measured at a wavelength of 510 nm using a linear equation to calculate the total flavonoid content of GD sample. The standard deviation (± SD) of three repeated samples was then calculated.

### Animals

A total number of 60 male imprinting control region mice (3 weeks old) were randomly distributed, with 5 mice per cage. The mice were taken from the Experimental Animal Centre, Soochow University. They were under standard laboratory conditions (18–22°C, humidity 50–80% with a cycle of 12–12-h light/dark) and allowed to feed and drink freely. After rearing with a normal diet for 5 days, the mice were randomly divided into four groups (*n*=15 each): normal group, diabetic model group, and two GD-treated diabetic groups at doses of 1.2% (L) and 4.8% (H). The normal group received normal chow, and the other groups were fed a high-fat diet (high-fat diet recipes: 18% lard, 20% sugar, 3% egg yolk, and 59% basal diet). After 10 days, STZ (Sigma-Aldrich Fine Chemicals, USA) was injected into the mice in the diabetic model group and two GD-treated diabetic groups through the caudal vein. Specifically, STZ (100 mg/kg.BW) was dissolved in cold 0.1-M citrate buffer (pH 4.4) that was always freshly prepared for immediate usage. After 1 week, tail vein fasting blood glucose (FBG) was measured, and mice with FBG levels ≥11.1 mmol/l were considered diabetic. Subsequently, mice in the normal group and diabetic model group were treated with a normal diet. The two GD-treated diabetic groups received different doses of GD diet daily (the diets with 1.2% and 4.8% GD, respectively). The protocols for all animals were prepared in accordance with the standards for laboratory animal care accepted by the Committee on Animal Experimentation and Ethics of Soochow University.

Over 4 weeks, the mice in each group were fed with food and water daily. The average weight of each group of mice was measured every 5 days. The FBG levels were measured using a ONETOUCH Blood Glucose Meter (LifeScan, Inc., Milpitas, CA, USA), once a week after fasting overnight. After 4 weeks of treatment, mice were fasted overnight and then sacrificed. The eyeballs of the mice were extracted, and blood was drawn. Their livers and pancreas were excised and weighed.

### Antioxidant activity and lipid peroxidation level

Antioxidant enzymes including glutathione peroxidase (GSH-Px) and total superoxide dismutase (T-SOD) activities in the liver tissue were tested using commercial kits (Nanjing Jiancheng Bioengineering Institute, China). Lipid peroxidation level of the liver tissue was determined according to the content of MDA generated. MDA level assay in the liver tissue was performed according to kit protocols (Nanjing Jiancheng Bioengineering Institute, China).

### Serum insulin level

The mouse serum insulin level was measured by mouse enzyme-linked immunosorbent assay kit (Nanjing Jiancheng Bioengineering Institute, China). All procedures were performed in accordance with the manufacturer's instructions.

### Blood lipid and liver glycogen content

The total cholesterol (CHOL), triglyceride (TG) and high-density lipoprotein cholesterol (HDL-C) levels were determined for assessing blood lipid level using BS-800 Chemistry Analyzer (Mindray Medical International Ltd., Shenzhen, China). The liver glycogen content was assayed by a glycogen assay kit (Nanjing Jiancheng Bioengineering Institute, China).

### QPCR

Total RNA was extracted from pancreatic tissues according to the Trizol RNA extraction method (TAKARA Biotechnology Co., Ltd., Dalian, China), and the concentration of RNA was quantified with a NanoDrop ND-1000 spectrophotometer (Thermo Scientific, USA). Synthesized cDNA was used for the qPCR. All primers (Table 1) were provided by ShineGene Co. (China). All quantifications were performed with beta-actin (β-actin) as the internal reference gene. Relative quantification of gene expression was analyzed using the ΔΔCT method. The qPCR conditions were 94°C for 4 min, followed by 35 cycles of 94°C for 20 s, 60°C for 30 s, and 72°C for 30 s; the signals were detected at 72°C.

**Table 1:** mRNA primer design of the key enzymes in pancreatic tissue

Gene	Primer sequences (5' to 3’)	Product length (bp)
*AKT-F*	TGTCTGCCCTGGACTACTTGC	166
*AKT-R*	GGCGTTCCGCAGAATGTC	166
*PI3K-F*	AAGCCATTGAGAAGAAAGGACTG	176
*PI3K-R*	ATTTGGTAAGTCGGCGAGATAG	176
*PDK-1-F*	CTGGGCGAGGAGGATCTG	132
*PDK-1-R*	CACAGCACGGGACGTTTC	132
*β-actin-F*	GAGACCTTCAACACCCCAGC	263
*β-actin-R*	ATGTCACGCACGATTTCCC	263

### Western blot analysis

Total proteins from pancreatic tissues were extracted and separated with a 10% sodium dodecyl sulfate-polyacrylamide gel electrophoresis (SDS-PAGE). Proteins were then transferred to polyvinylidene difluoride membranes (Millipore, Shanghai, China). The membranes were first blocked with 5% defatting milk for 2 h at room temperature and then incubated with monoclonal antibodies (Borson Biological Company, Beijing, China) against PDK-1(1:1,000), PI3K(1:1,000), AKT(1:1,000), phosphorylated AKT (P-AKT) (1:1,000) and GAPDH (1:3,000) overnight at 4°C. After being washed, the membranes were incubated with an appropriate secondary antibody (1:5,000) for 1.5 h at room temperature. Bands were visualized using a UVP detection system. Band intensity was quantified using LABWORKS 4.6.

### Statistical analysis

The data were expressed as the mean±standard deviation (±SD). Differences between two sets of data were evaluated using one-way ANOVA with the Origin 7.5 software program; *p*-values less than 0.05 were considered statistically significant.

## Results

### Total polysaccharides and flavonoids in GD

Polysaccharides and flavonoids are functional components of GD. Glucose and rutin are often used as standards for the determination of the content of total polysaccharides and flavonoids, respectively. After glucose and rutin standard curves were gained, the content of total polysaccharides and flavonoids of GD can be calculated according to the respective absorbance values. The linear regression equation of glucose standard curve was *y*=0.03284+15.79431*x* (*R* ^2^=0.99). According to the regression equation, the total polysaccharides content in the GD was 26.69±0.53 mg/g, and the linear regression equation of rutin standard curve was *y*=−0.00138+0.75567*x* (*R* ^2^=0.99). According to the regression equation, the total flavonoids content in the GD was 33.59±2.64 mg/g.

### Effect of GD on the weight of diabetic mice

**Fig. 1:**
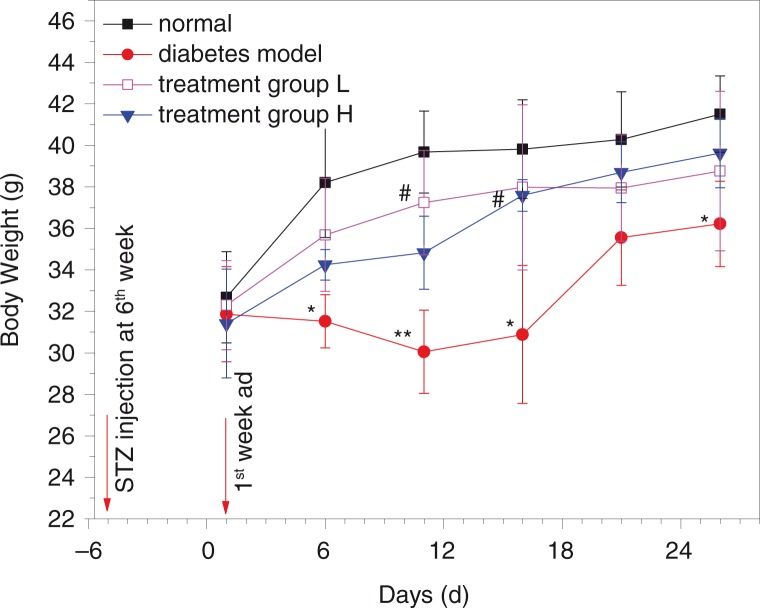
Effect of GD on the body weight of diabetic mice 7–10 weeks old. ad means oral administration. Treatment group L: 1.2% GD-treated diabetic group; treatment group H: 4.8% GD-treated diabetic group. Data are expressed as mean±SD. *p<0.05 and **p<0.01, respectively, versus the normal group; ^#^ *p*<0.05 versus the diabetic model group.

The changes in the body weights of mice are shown in Fig. 1. Compared with the normal group, high-fat diet and STZ-induced diabetic mice showed polyuria, polyphagia, and weight loss. The body weights increased slowly in the normal group during the 4 weeks, while the weights decreased slightly in the diabetic model group during the first 11 days and then increased slowly. The weight changes in the two GD-treated diabetic groups were similar to that of the normal group.

### Effect of GD on the FBG of diabetic mice

**Fig. 2:**
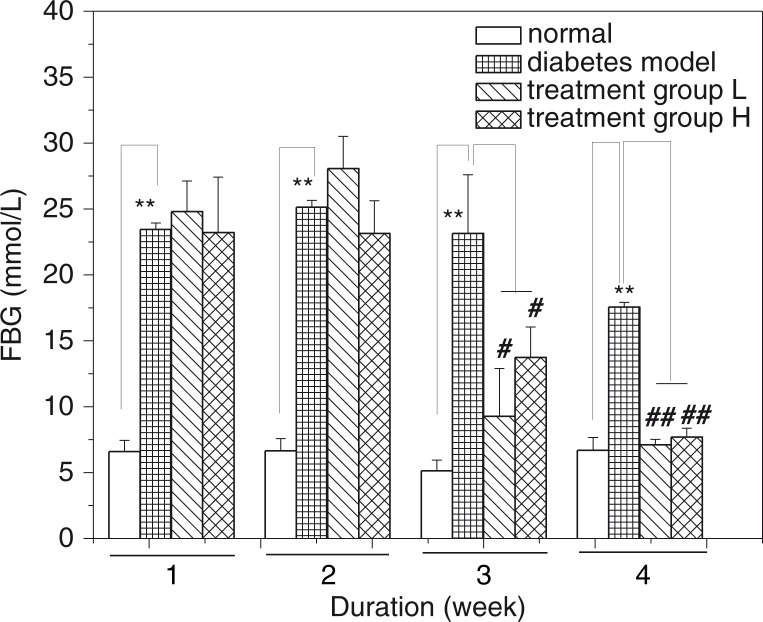
Effect of GD on FBG levels of diabetic mice. FBG: fasting blood glucose; treatment group L: 1.2% GD-treated diabetic group; treatment group H: 4.8% GD-treated diabetic group. Data are expressed as mean±SD. **p<0.01, versus the normal group; ^#^ *p*<0.05 and ^##^ *p*<0.01, respectively, versus the diabetic model group.

The changes in FBG level of mice are shown in Fig. 2. FBG levels in diabetic model mice were always significantly different compared with the normal mice (*p*<0.01). And no obvious changes were observed with FBG level in the first and second weeks. However, there is a significant reduction in FBG level with continual GD treatment in the third week (*p*<0.05) and fourth week (*p*<0.01), relative to that of the diabetic model group. Overall, after 4 weeks of GD treatment, the FBG levels of diabetic mice were obviously reduced so that their blood glucose values were closer to that of the normal group. These results suggested that GD can effectively reduce the FBG levels of high-fat diet and STZ-induced diabetic mice.

### Effect of GD on the antioxidant capacity of diabetic mice

Table 2 reveals the liver damage in the high-fat diet and STZ-induced diabetic mice. The GSH-Px level of diabetic model mice decreased significantly compared with the normal mice (*p*<0.01). After 4 weeks of treatment with different doses of GD, the GSH-Px level increased in both treatment groups L and H. In addition, the GSH-Px level in treatment group H increased significantly and was obviously different compared with the diabetic model group (*p*<0.01), while the GSH-Px level in treatment group L was not significantly different compared with the model group.

**Table 2:** Effects of GD on the GSH-Px as well as T-SOD activities and MDA levels in mice

Groups	Dose	GSH-Px (U/mgprot)	T-SOD (U/mgprot)	MDA (nmol/mgprot)
Normal	—	1216.84±7.33	100.29±0.52	0.47±0.09
Diabetes model	—	694.87±165.24**	63.01±0.12**	1.04±0.01**
Experimental	Treatment group L	733.75±4.24	95.55±0.73##	0.52±0.09##
	Treatment group H	1145.65±6.00##	96.67±10.80#	0.67±0.21#

Treatment group L: 1.2% GD-treated diabetic group; Treatment group H: 4.8% GD-treated diabetic group. Data are mean±SD.

** *p*<0.01 versus the normal group;

# *p*<0.05 and

## *p*<0.01, respectively, versus the diabetic model group.

The T-SOD level in diabetic model mice decreased significantly compared to the normal group (*p*<0.01). After 4 weeks of treatment with different doses of GD, the T-SOD levels in treatment groups L and H increased significantly, respectively, compared with the diabetic model group (*p*<0.01 and *p*<0.05).

The MDA level of diabetic model mice was higher than the normal group (*p*<0.01). After different doses of GD treatment, the MDA level of treatment group H decreased compared with the model group (*p*<0.05). Furthermore, there was a significant difference in MDA levels between treatment group L and the model group (*p*<0.01).

Based on these results, we conclude that the antioxidant capacity could be enhanced in diabetic mice by GD treatment.

### Effect of GD on the serum insulin level in diabetic mice

**Fig. 3:**
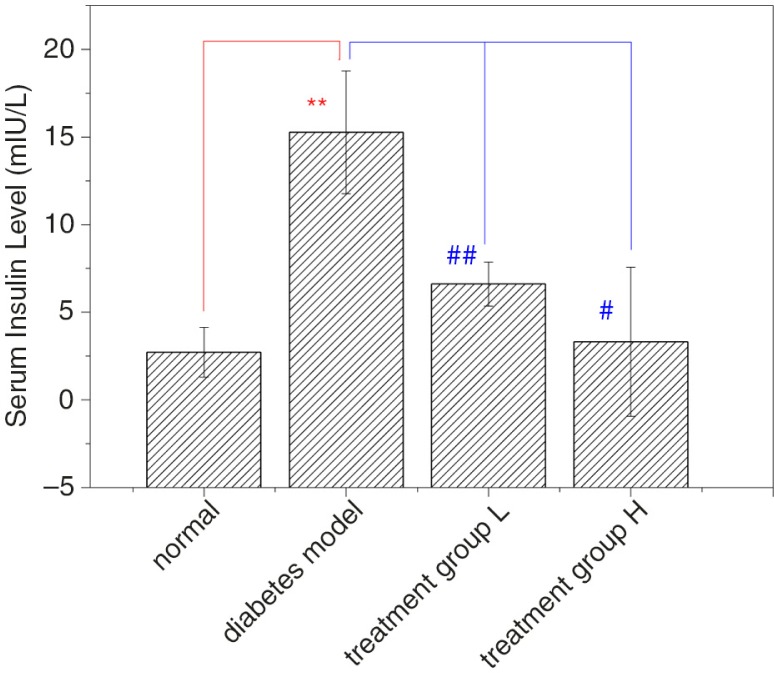
Effect of GD on the serum insulin level in diabetic mice. After 4 weeks of GD treatment in high-fat diet and STZ-induced type 2 diabetic mice, the serum insulin level was measured in mice during the fasting state. Treatment group L: 1.2% GD-treated diabetic group; treatment group H: 4.8% GD-treated diabetic group. Data are expressed as mean±SD. **p<0.01, versus the normal group; ^#^ *p*<0.05 and ^##^ *p*<0.01, respectively, versus the diabetic model group.

As shown in Fig. 3, the serum insulin levels in diabetic model mice were higher than the normal mice (*p*<0.01). After 4 weeks of treatment with different doses of GD, the serum insulin levels in treatment groups L and H decreased significantly compared with the diabetic model group (*p*<0.01 and *p*<0.05). Thus, GD treatment can reduce insulin levels and ameliorate insulin resistance in diabetic mice.

### Effects of GD on blood lipids and liver glycogen in diabetic mice

Diabetes is always accompanied by high cholesterol, high triglycerides, lipoprotein abnormalities and other complications. Liver glycogen synthesis and degradation are controlled by insulin. Diabetes may be caused by a decrease in liver glycogen synthesis and increase in liver glycogen degradation. Therefore, the determination of blood lipids and liver glycogen levels is of great significance for the treatment of diabetes.

Table 3 showed that the TG and CHOL levels of diabetic model mice were much higher than the normal mice. After 4 weeks of GD treatment, the TG and CHOL levels in treatment groups L and H were lower than the diabetic model group.

**Table 3:** Effect of GD on blood lipids and liver glycogen levels in diabetic mice

Groups	Dose	TG (mmol/L)	CHOL-C (mmol/L)	HDL-C (mmol/L)	Liver glycogen (mg/g)
Normal	—	1.36±0.12	2.85±0.08	2.13±0.30	5.36±1.25
Diabetes model	—	1.87±0.77	3.14±0.55	1.94±0.30	1.64±0.26*
Experimental	Treatment group L	1.27±0.57	2.74±0.20	1.83±0.43	3.41±1.07
	Treatment group H	1.53±0.52	2.91±0.22	2.26±0.43	2.31±0.57

Treatment group L: 1.2% GD-treated diabetic group; Treatment group H: 4.8% GD-treated diabetic group. Data are mean±SD.

* *p*<0.05 versus the normal group.

The serum HDL-C level in diabetic model mice was lower than normal mice. After different doses of GD treatment, the serum HDL-C level in treatment group H was higher than the diabetic model group.

The measurements of liver glycogen showed that the liver glycogen content in diabetic model mice was markedly lower than normal mice (*p*<0.05). The content of liver glycogen in treatment groups L and H was higher than the diabetic model group after 4 weeks of GD treatment.

Overall, all these results revealed the benefits of GD on the amelioration of blood lipids and liver glycogen levels in diabetic mice.

### Gene expression and protein expression of the AKT/PI3K signaling pathway

**Fig. 4:**
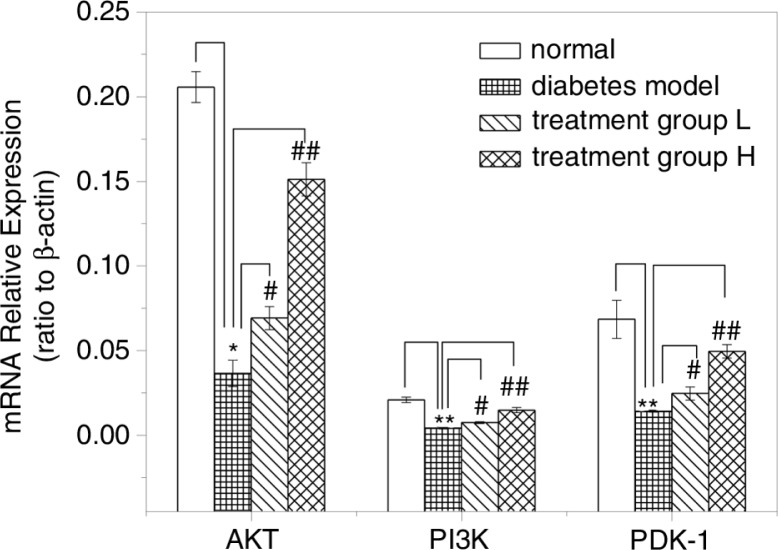
Effect of GD on AKT, PI3K and PDK-1 mRNA expressions in pancreatic tissues of diabetic mice. The mRNA relative expressions of AKT, PI3K, and PDK-1 were tested using qPCR. Treatment group L: 1.2% GD-treated diabetic group; treatment group H: 4.8% GD-treated diabetic group. Data are mean±SD. *p<0.05 and **p<0.01, respectively, versus the normal group; ^#^ *p*<0.05 and ^##^ *p*<0.01, respectively, versus the diabetic model group.

AKT/PI3K is an important signaling pathway downstream of the insulin receptor. The AKT/PI3K signaling pathway is closely related to the development of diabetes and the mechanism of decreased insulin sensitivity (23, 24). To determine the changes in the AKT/PI3K signaling pathway in mice of different groups, the present study used qPCR to detect the gene expression levels of AKT, PI3K and PDK-1 in pancreatic tissue. Figure 4 shows that the gene expression levels of AKT, PI3K and PDK-1 in diabetic model mice were much lower than normal mice. After 4 weeks of GD treatment, the gene expression levels (AKT, PI3K and PDK-1) in treatment groups L and H were all improved compared to the diabetic model mice. The results showed that GD treatment can increase the gene expression levels of AKT, PI3K and PDK-1 in diabetic mice. Therefore, we may conclude that GD had a positive effect on the AKT-PI3K pathway.

**Fig. 5:**
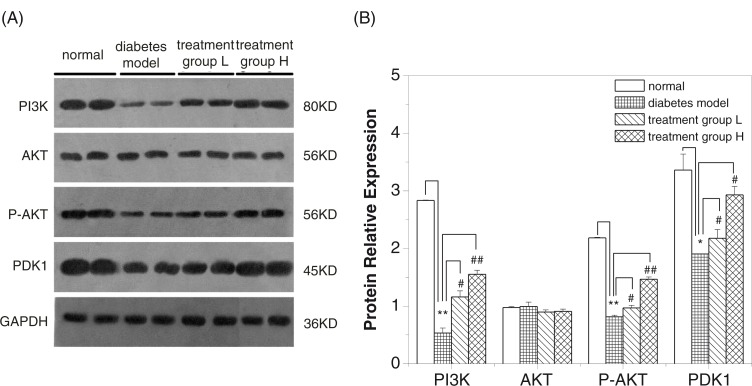
Effect of GD on AKT, P-AKT, PI3K, and PDK-1 protein expressions in pancreatic tissues of diabetic mice. (A) Western blot analysis of AKT, P-AKT, PI3K, and PDK-1 protein expressions. (B) Quantitative analysis of AKT, P-AKT, PI3K and PDK-1 protein expressions. Treatment group L: 1.2% GD-treated diabetic group; Treatment group H: 4.8% GD-treated diabetic group. Data are given by mean±SD. *p<0.05 and **p<0.01, respectively, versus the normal group; ^#^ *p*<0.05 and ^##^ *p*<0.01, respectively, versus the diabetic model group.

Furthermore, suppression of insulin signaling pathway with diabetes was confirmed by western blotting analysis of some components in AKT-PI3K pathway (Fig. 5a). We found that the protein expression levels of PI3K and PDK-1 were significantly increased in treatment groups L and H, which were essentially in agreement with that of the gene expression levels in the AKT/PI3K signaling pathway (Fig. 5b). Interestingly, there were no statistical difference in total AKT proteins (Fig. 5b). However, phosphorylated AKT (p-AKT) was reduced significantly with diabetes, and GD exerted a significantly enhancing effect on AKT phosphorylation (Fig. 5b). Therefore, this assay further revealed that the hypoglycemic effect of GD may be achieved by activating the AKT/PI3K signaling pathway during diabetes.

## Discussion

Most traditional Chinese medicines treat disease by synergy gained from natural materials. Some investigations have found that GD contains many hypoglycemic ingredients that may have the potential to treat diabetes. It has been reported that phenolics and flavonoids in GD are of antioxidant activity and free radical-scavenging capacity, which improves antioxidant potential in diabetic mice (25). Additionally, the positive effects of polyphenols on glucose homeostasis observed in a large number of *in vitro* and animal models are supported by epidemiological evidence on polyphenol-rich diets. And growing evidence suggests the hypoglycemic activity of flavonoid compounds in GD (12, 17, 26), including quercetin, isoquercitrin, rutin, and kaempferol-3-*O*-rutinoside. Furthermore, polysaccharides from GD have been demonstrated exerting an anti-diabetic effect (15). Chou et al. reported that these hypoglycemic constituents of GD are fructooligosaccharides, including beta-d-fructofuranose, sucrose, 1-kestose, nystose, and 1^F^-beta-fructofuranosylnystose (27).

Type 2 diabetes, which is the major type in the diabetic population, is characterized by hyperglycemia, hyperlipidemia and insulin resistance (28). In the present study, a type 2 diabetic mouse model was established by a high-fat diet and STZ injection. This method has low toxicity and allows a high-animal-survival rate. Low-dose intravenous injection of STZ can destroy islet β cells or cause islet β cell necrosis in mice, and STZ in conjunction with a high-fat diet can cause animals to exhibit the natural progression and metabolic characteristics of type 2 diabetic patients. Therefore, this method has become the main method for the establishment of diabetic mice models (29). Based on these findings, we successfully induced the pathogenesis of type 2 diabetes and insulin resistance in mice. In this study, insulin sensitivity in untreated diabetic mice was significantly damaged. The mice in the diabetic group showed severe metabolic abnormalities, including elevated blood glucose, TG and CHOL levels, and insulin resistance.

Insulin resistance can cause disorders of glucose metabolism and lipid metabolism, which could increase the free fatty acid triglyceride and cholesterol levels in plasma (30). Furthermore, too much insulin leads to decreased target sensitivity of insulin, which weakens the ability of blood glucose to be stored by insulin-sensitive tissues. Our results clearly indicated that GD can significantly ameliorate insulin secretion in diabetic mice.

The liver is a major organ in the glucose metabolic response to insulin (31). Some researchers have reported impaired glycogen storage in the liver of diabetic animals. Our results showed that GD-lyophilized powders significantly decreased the FBG levels of diabetic mice, and its effect was better than that of the GD water extract reported by Ma et al. (32). Furthermore, the GD powder could enhance glycogen synthesis and glucose metabolism in the liver.

Oxidative stress is elevated in individuals with type 2 diabetes and may be the common pathway for insulin resistance and metabolic syndrome in type 2 diabetes (33). Obesity and a high-fat diet may be the main reasons underlying oxidative stress (34). The liver is the main target of insulin and the key organ of glucose metabolism; however, it is also the main organ involved in detoxifying processes and is frequently exposed to damage from oxidative stress. SOD and GSH-Px are the primary scavenger enzymes that participate in detoxifying reactive oxygen species (ROS) in mice. The role of SOD is to catalyze harmful oxygen free radicals in hydrogen peroxide (H_2_O_2_) and oxygen (O_2_) and reduce the intracellular concentration of superoxide (35). The function of GSH-Px is to catalyze lipid hydroperoxides in their corresponding alcohols and to catalyze free hydrogen peroxide to H_2_O (36). In our experiment, treatment with GD significantly reduced the level of MDA in diabetic mice. These results indicated that GD is effective in improving antioxidant activity and reducing organ damage caused by oxidative stress.

To investigate the effects of GD on insulin sensitivity, the expression levels of mRNA and protein in the pancreatic insulin signaling pathway were determined. The AKT/PI3K pathway plays a vital role in insulin signal transduction and is a classical glucose metabolism pathway mediated by insulin. PI3K is a key protein that transmits insulin signals to regulate glucose metabolism. Downstream signaling molecules of PI3K include 3-inositol phosphate (PIP3) dependent protein kinase (PDK) and AKT. PI3K and AKT are also known to play a role in glucose transporter 4(GLUT4) translocation. In this experiment, the AKT/PI3K signaling pathway was activated in GD-treated type 2 diabetic mice. Although the expression of AKT mRNA was improved by GD treatment, AKT protein levels remained unchanged. This situation would indicate a translational control mechanism regulating the AKT amount independent of its mRNA contents. In addition, AKT was affected by diabetes in its phosphorylation form because p-AKT contents were reduced, which are in parallel with the mRNA expressions. The above post-translational mechanism leading to the phosphorylation of AKT was shown to act in parallel with the mRNA expressions (37).

This study is novel because the leaves and stems of GD were freeze dried to retain the plant's physicochemical properties and physiological activity to maintain as much of the plant's active chemicals as possible. The principle underlying this method involves removing moisture from GD by evaporation and sublimation to dehydrate the material. In this study, the direct utilization of vacuum freeze-drying technology was more economical than the traditional method and still retained the active ingredients of GD. However, the traditional methods of water or ethanol extraction reported in previous studies may lead to the loss or inactivation of active ingredients. Our GD powder contained 26.69±0.53 mg/g of crude polysaccharides and 33.59±2.64 mg/g of the total flavonoids, which were higher than the respective reported concentrations by Wang et al. (6) and Jiang et al. (38). Therefore, our study further demonstrated that lyophilizing technology can preserve the active ingredients of GD more effectively than other methods.

Our experiment demonstrated the hypoglycemic effect of GD on diabetes. However, some limitations of our study should be noted. First, the type 2 diabetes mouse model was established using high-fat feeding and low-dose STZ in our study. Although this model shares many metabolic abnormalities that are present in patients, it still may have some limited relevance to clinical conditions. Second, the present study was designed to investigate the effects of lyophilized GD powders in diabetes for the first time and was only a preliminary study. As such, some imperfections can hardly be avoided. Therefore, further study about the detailed mechanism of hypoglycemic activities of GD will be performed.

In conclusion, the administration of lyophilized GD powders can effectively improve glucose metabolism and insulin resistance in mice with type 2 diabetes induced by administration of a high-fat diet and STZ injection. In addition, the GD treatment mitigates oxidative stress, which may be the potential mechanism underlying improved glucose metabolism and insulin resistance. Therefore, GD is likely to be an effective medicine for the treatment of diabetes in the future.

## Conclusions

The total polysaccharides content of GD was 26.69±0.53 mg/g, and the total flavonoids content was 33.59±2.64 mg/g. The use of the lyophilization technique preserved the plant's physicochemical properties and physiological activity, and the concentration of active ingredients was significantly higher than other reports that used water or alcohol extraction. In addition, the weight loss in high-fat diet and STZ-induced diabetic mice was ameliorated by GD treatment. Furthermore, the FBG and insulin levels were effectively reduced, and the antioxidant capacity and dyslipidemia were ameliorated in diabetic mice after GD treatment, which had protective effects against diabetes-induced liver damage, and the molecular biology results showed that GD has an obvious effect on reducing FBG by activating the AKT/PI3K signaling pathway. Therefore, these findings provide evidence supporting the application of GD as a preventive and therapeutic measure for type 2 diabetes and insulin resistance.
